# Retinoic Acid Is Required for Oligodendrocyte Precursor Cell Production and Differentiation in the Postnatal Mouse Corpus Callosum

**DOI:** 10.1523/ENEURO.0270-19.2019

**Published:** 2020-01-06

**Authors:** Vivianne E. Morrison, Victoria N. Smith, Jeffrey K. Huang

**Affiliations:** 1 Department of Biology and Center for Cell Reprogramming, Georgetown University, Washington, DC 20057; 2 Interdisciplinary Program in Neuroscience, Georgetown University, Washington, DC 20057

**Keywords:** meninges, oligodendrocyte precursor cells, oligodendrocytes, postnatal, retinoic acid, subcortical white matter

## Abstract

Myelination of the CNS relies on the production and differentiation of oligodendrocyte (OL) precursor cells (OPCs) into mature OLs. During the first month of postnatal life, OPCs that populate the corpus callosum (CC) arise from neural stem cells (NSCs) in the subcallosal subventricular zone (SVZ), and then differentiate to generate myelinating OLs. However, the signals that regulate these processes are not fully understood. In this study, we show that endogenous expression of the retinoic acid (RA)-synthesizing enzyme retinaldehyde dehydrogenase 2 (RALDH2) is required for OPC generation and differentiation in the postnatal subcortical white matter. In male and female pups, conditional deletion of *Raldh2* reduced OPC numbers and differentiation. Moreover, decreased OPC numbers coincided with reductions in NSC survival and expression of the sonic hedgehog (SHH) signaling effector protein *Gli1* in the SVZ. Additionally, GFAP expression in the CC was decreased, and cortical neuron numbers were altered. Our work suggests a role for endogenous RALDH2-dependent RA synthesis in OPC production and differentiation in the CC, as well as in the development of other cell types derived from NSCs in the embryonic ventricular zone (VZ) and SVZ, as well as the postnatal subcallosal SVZ.

## Abstract (precis)

Myelination of the CNS relies on the production and differentiation of oligodendrocyte (OL) precursor cells (OPCs) into mature OLs. During the first month of postnatal life, OPCs that populate the corpus callosum (CC) arise from neural stem cells (NSCs) in the subcallosal subventricular zone (SVZ), and then differentiate to generate myelinating OLs. However, the signals that regulate these processes are not fully understood.

## Significance Statement

Postnatal forebrain myelination requires the production of oligodendrocytes (OLs), but the signals ensuring normal numbers of OLs have not been fully described. We demonstrate that loss of the retinoic acid (RA)-synthesizing enzyme retinaldehyde dehydrogenase 2 (RALDH2) decreased the number and differentiation of OL precursor cells (OPCs), leading to a deficit in OLs. These reductions co-occurred with increased death of neural stem cells (NSCs), reduced expression a sonic hedgehog (SHH) pathway effector, and altered development of NSC-derived neurons and white matter astrocytes. Our results suggest that endogenous RA synthesis is required for the development of NSC-derived forebrain cells.

## Introduction

In the CNS, oligodendrocytes (OLs) promote neuronal survival (Wilkins et al., 2003; Chang et al., 2016), regulate neuronal energy metabolism (Saab et al., 2013), and influence neurotransmission through myelination (Nave, 2010). In the postnatal corpus callosum (CC), >80% of OLs arise from differentiating dorsally derived OL precursor cells (OPCs), which are characterized in part by their expression of the proteoglycan neural/glial antigen 2 (NG2) and arise from neural stem cells (NSCs) in the subcallosal subventricular zone (SVZ). This process depends on sonic hedgehog (SHH) signaling in NSCs (Richardson et al., 2006; Winkler et al., 2018). Given the role of OLs in postnatal brain function and devastating effects of demyelination (Steenweg et al., 2010; Stangel, 2012), it is important to identify signals that support OPC production and differentiation.

One candidate is retinoic acid (RA), a highly conserved morphogen synthesized from dietary vitamin A. Vitamin A (also called retinol) undergoes two oxidative steps, the second of which is mediated by retinaldehyde dehydrogenases (RALDH; Napoli, 2012). RA binds to nuclear RA receptor (RAR)-retinoid X receptor (RXR) heterodimers at RA response elements (RAREs) and modifies chromatin to regulate gene transcription (Paschaki et al., 2013). In this way, RA influences fundamental processes in CNS cells, such as proliferation, survival, differentiation, and maturation (Maden, 2002; Gudas, 2012).

Early studies suggested a link between vitamin A deficiency and myelination *in vivo* (Clausen, 1969; Kean, 1970; Bhat and Rao, 1978). Later, *in vitro* studies found that exogenous RA influences OPC differentiation (Barres et al., 1994; Laeng et al., 1994; Noll and Miller, 1994). Moreover, it was found that RA signaling supports OPC differentiation and remyelination following spinal cord injury, and expression of RALDH2 in NG2^+^ cells was necessary for this effect (Huang et al., 2011; Goncalves et al., 2019). Critically, in the embryonic forebrain of *Raldh2* null mice, transcription factors and signaling pathways known to promote OPC production (i.e., OLIG2 and SHH, respectively) were reduced (Ribes et al., 2006; Emery, 2010; Tong et al., 2015), raising the possibility of a role for RALDH2-dependent endogenous RA synthesis in OL development. However, since *Raldh2* null mice die *in utero*, whether this occurrs *in vivo* remained unknown.

RALDH2 expression patterns in the postnatal brain are not fully understood. It is well accepted that RALDH2 is expressed in the meninges (Smith et al., 2001; Wagner et al., 2002; Siegenthaler et al., 2009; Haushalter et al., 2017), and it is likely that cells in the parenchymal neurovascular niche express RALDH2, but the exact identity of RALDH2^+^ cells is unclear: some report co-localization with NG2 (Mey et al., 2005; Kern et al., 2007) while others find *Ng2* and *Raldh2* to be largely mutually exclusive and expressed in different perivascular cell populations: *Ng2* in mural cells [pericytes and smooth muscle cells (SMCs)] and RALDH2 in a subset of perivascular cells with fibroblast-like properties (FB cells), characterized by expression of collagen, type 1, α1 (Col1a1; Kelly et al., 2016; Vanlandewijck et al., 2018). However, both of these studies show that, irrespective of NG2 status, cells expressing RALDH2 are positive for platelet-derived growth factor receptor β [PDGFRβ; in addition to consulting the protein expression data from Kelly et al. (2016), the online gene expression database generated by Vanlandewijck et al. (2018), was used to make this determination]. Finally, RALDH2 has been observed to co-localize with mature OL markers like RIP and CNPase in the adult spinal cord (Mey et al., 2005), showing that OLs derived from NG2^+^ OPCs express RALDH2 in the CNS.

To determine whether endogenous RA synthesis impacts postnatal OL development, we conditionally deleted *Raldh2* in the CNS from cells that express or have expressed NG2 at some point in their lineage. We found that the numbers of OPCs and OLs in the postnatal CC were reduced in the *Raldh2* cKO, and the deficit in OL lineage cells was accompanied by increased NSC death and reduced expression of a downstream effector of the SHH pathway in the subcallosal SVZ. Additionally, we observed altered development of callosal astrocytes and cortical neurons in cKO mice. Our results suggest that endogenous RALDH2-dependent RA synthesis regulates the generation of multiple forebrain cell types and the maturation of OL lineage cells.

## Materials and Methods

### Experimental design and statistical analysis

Comparisons were made between *Ng2-Cre:Raldh2^flox/flox^* mice and *Raldh2^flox/flox^* control littermates. In some cases, comparisons were made between time points within genotypes. Males and females were equally represented in the analyses. Tissue samples were collected as litters became available over a period of several months. The experimenter was blinded to the genotypes and time points until all the raw values (i.e., cell number, puncta number, and area) were recorded in Excel. For each experiment, there were at least three mice per genotype per time point. For each mouse in an experiment, nine images were analyzed (three images per brain section, three brain sections per slide). Each experiment was independently repeated at least twice using different animals for each round. Data from multiple independent experiments were collated after ensuring that variations in the means were not due to inter-experiment variation through a MANOVA. GraphPad Prism version 8 (RRID:SCR_002798) was used to perform all statistical analyses, including one-tailed and two-tailed unpaired *t* tests, one-way ANOVAs with Sidak’s test for multiple comparisons, and Shapiro–Wilk normality tests. Exact *p* values are reported when possible, but when not supplied by Prism the following notations are used to indicate the significance: *p < 0.05, **p < 0.01, ***p < 0.001, ****p < 0.0001. All comparisons made in this work are included in a statistical summary (Table 1), indicating the groups and time points compared, the data distribution, the test used, and the confidence intervals.

**Table 1:** Statistical summary

Identifier	Shapiro–Wilk (α = 0.05)	Test	95% CI
a	Normal	*Raldh2* mRNA puncta/mm; unpaired *t* test between genotypes at P2, one-tailed	–2521 to –1732
b	Normal	PDGFRβ^+^ structures/mm^2^; unpaired *t* test between genotypes at P14, two-tailed	–42.62 to 16.82
c	Normal	Laminin^+^ structures/mm^2^; unpaired *t* test between genotypes at P14, two-tailed	–94.79 to 21.25
d	Normal	PDGFRβ^+^Laminin^+^/Laminin^+^ structures %, unpaired *t* test between genotypes at P14, two-tailed	–2.765 to 21.69
e	Normal	OLIG2^+^ cells/mm^2^; one-way ANOVA with Sidak’s test between genotypes at individual time points	
		P2	4.080 to 494.0
		P7	80.47 to 547.6
		P14	126.6 to 593.7
		P21	108.9 to 681.0
f	Normal	OLIG2^+^ cells/mm^2^; unpaired *t* test within control genotype between individual time points P2 vs P21, one-tailed	–441.0 to 282.2
g	Normal	OLIG2^+^ cells/mm^2^; unpaired *t* test within cKO genotype between individual time points P2 vs P21, one-tailed	–490.0 to 39.44
h	Normal	PDGFRα^+^OLIG2^+^ cells/mm^2^; one-way ANOVA with Sidak’s test for multiple comparisons between genotypes at individual time points	
i	Normal	PDGFRα^+^OLIG2^+^ cells/mm^2^; one-way ANOVA with Sidak’s test for multiple comparisons within control genotype at individual time points	
		P2 vs P7	–121.2 to 55.21
		P7 vs P14	–184.2 to –16.61
		P14 vs P21	8.089 to 221.1
		P2 vs P21	–224.6 to –42.19
j	Normal	PDGFRα^+^OLIG2^+^/OLIG2^+^ cells/mm^2^; one-way ANOVA with Sidak’s test for multiple comparisons between genotypes at individual time points	
		P2	–26.25 to –9.080
		P7	–21.13 to –3.288
		P14	–20.19 to –1.376
		P21	–12.09 to 6.720
k	Normal	CC1^+^OLIG2^+^ cells/mm^2^; one-way ANOVA with Sidak’s test for multiple comparisons between genotypes at individual time points	
		P7	–21.38 to 87.30
		P14	32.72 to 141.4
		P21	49.38 to 158.1
l	Normal	CC1^+^OLIG2^+^ cells/OLIG2^+^ cells %; one-way ANOVA with Sidak’s test for multiple comparisons between genotypes at individual time points	
		P14	2.156 to 20.30
		P21	1.024 to 19.16
m	Normal	PLP^+^ area (mm^2^); one-way ANOVA with Sidak’s test for multiple comparisons between genotypes at individual time points	
		P7	–18.20 to 107.8
		P14	30.06 to 138.5
		P21	25.44 to 139.2
		P120	69.15 to 211.4
n	Normal	TUNEL^+^ cells/mm^2^; unpaired *t* test within control genotype between individual time points P2 vs P7, one-tailed	–41.86 to –15.94
o	Normal	TUNEL^+^ cells/mm^2^; unpaired *t* test within cKO genotype between individual time points P2 vs P7, one-tailed	–18.13 to 4.871
p	Normal	TUNEL^+^ cells/mm^2^; one-way ANOVA with Sidak’s test for multiple comparisons between genotypes at individual time points	
		P2	–9.109 to 16.03
		P7	–30.05 to –7.565
		P14	0.9951 to 10.17
q	Normal	IBA1^+^ cells/mm^2^ one-way ANOVA with Sidak’s test for multiple comparisons between genotypes at individual time points	
		P7	–46.17 to 51.34
		P14	–92.57 to 4.941
		P21	–37.76 to 67.56
r	Normal	*Gli1* mRNA puncta/mm^2^; unpaired *t* test between genotypes at P2, one-tailed	–15,405 to –2876
s	Normal	*Gli1* mRNA puncta/cell; n= number of cells, unpaired *t* test between genotypes at P2, one-tailed	–3.692 to –1.703
t	Normal	*Gli1^+^Olig2^+^* cells/mm^2^; unpaired *t* test between genotypes at P2, one-tailed	–53.01 to –33.57
u	Normal	ÄGFAP^+^ area (mm^2)^; one-way ANOVA with Sidak’s test for multiple comparisons between genotypes at individual time points	
		P2	0.8137 to 7.880
		P7	4.695 to 12.44
		P14	6.558 to 14.30
		P21	3.638 to 11.38
v	Normal	SATB2^+^ cells/mm^2^; unpaired *t* test between genotypes at P2, one-tailed	159.9 to 1991
w	Normal	CTIP2^+^ cells/mm^2^; unpaired *t* test between genotypes at P2, two-tailed	–172.6 to –68.02
x	Normal	TBR1^+^ cells/mm^2^; unpaired *t* test between genotypes at P2, two-tailed	–1407 to –439.5
y	Normal	Cortical thickness (um); unpaired *t* test between genotypes at P2, two-tailed	–234.6 to 26.12

### Animals

Mice of both sexes from the following strains were used: the B6;FVB-Ifi208Tg(Cspg4-cre)1Akik/J line (RRID:IMSR_JAX:008533; Zhu et al., 2008) and the floxed *Raldh2* line (*Raldh2*^L2/L2^, here written as *Raldh2*^fl/fl^; Vermot et al., 2006). The *Raldh2*^L2/L2^ line was generously provided by Dr. Wojciech Krezel and Dr. Pascal Dollé (IGBMC, Strasbourg, France). Mice were maintained on a 12/12 h light/dark cycle with food and water *ad libitum*. All experiments were performed in accordance with approved Institutional Animal Care and Use Committee (IACUC) protocols of Georgetown University.

### Genotyping primers

Primers were synthesized by Eurofins Genomics, Inc. (https://www.eurofinsgenomics.com/en/products/dnarna-synthesis/all-oligo-options/). Primer sequences: NG2-Cre, product size: 100 bp, Tm: 55°C; forward: GCGGTCTGGCAGTAAAAACTATC; reverse: GTGAAACAGCATTGCTGTCACTT; control (IL-2), product size: 324 bp, Tm: 55°C; forward: CTAGGCCACAGAATTGAAAGATCT; reverse: GTAGGTGGAAATTCTAGCATCATCC; RALDH2^fl/fl^, product size: WT, 150 bp; L2: 250 bp, Tm: 61°C; forward: CCCTCCGCTTGTCAAACCACCTTCTGCTATATT; reverse: GGCAGCTGTGGAGAACATTTTATATCCATGTTG.

### Sample collection and processing

Full litters were collected at postnatal day (P)2, P7, P14, P21, and P120. Animals were killed with isoflurane before cervical dislocation and decapitation. The brain was extracted, embedded in OCT, and flash-frozen in ice-cold isopentane, then stored at –80°C.

### Sectioning

**Figure 1.**
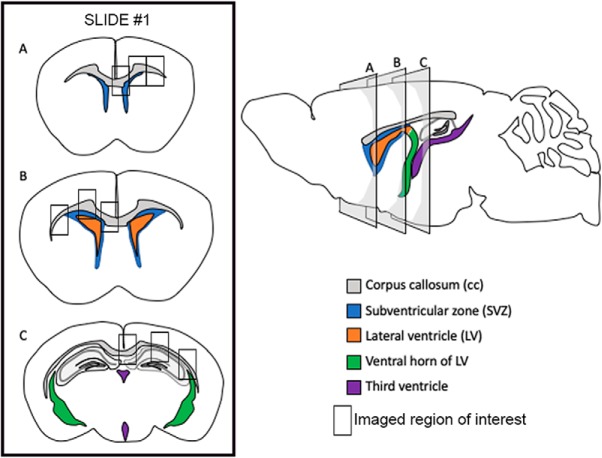
Illustration of sample collection and sectioning. Representative slide with three coronal sections (left, sections ***A–C***) that correspond to three regions along the rostral-caudal axis depicted on the sagittal diagram of the mouse brain (right, regions ***A–C***). A color-coded key indicates which structures on the coronal and sagittal sections are corresponding. The slide on the left also shows three small boxes on each section that correspond to regions imaged and used for analysis. Right and left sides were counterbalanced as shown.

The whole-brain samples embedded in OCT were left for 1 h in the cryostat (–20°C) to soften before cryosectioning. The collection of 10-μm-thick sections began when the hypercellular region of the SVZ appeared under the anterior forceps of the CC. Thirty slides labeled #1 through #30, each bearing three sections corresponding to different points along the rostral-caudal axis (Fig. 1), were collected for each animal and the remaining OCT-embedded brain was returned to –80°C. The sections were stored at –80°C until use.

### Immunofluorescence

Slides were removed from the –80°C freezer and immediately fixed in 4% paraformaldehyde (PFA) for 30 min at room temperature. Slides were washed twice in 1× Tris-buffered saline (TBS) for 5 min, permeabilized for 2 min in 1% Triton X-100 in 1× TBS and blocked with 10% donkey serum (Sigma-Aldrich D9663), 0.25% Tween in 1× TBS for 30 min at room temperature. Primary antibodies were diluted in 1× TBS (see below for dilution factors) and incubated overnight at 4°C. Slides were washed twice for 3 min in 0.05% Tween in 1× TBS before incubation at room temperature in the dark for 1 h with the secondary antibody diluted 1:1000 in 1× TBS. Slides were washed twice for 3 min in 0.05% Tween in 1× TBS in an opaque slide rack. ProLong Gold Antifade mounting media with DAPI was applied before placing a coverslip over the sections. Slides dried overnight at room temperature in the dark before imaging. Negative controls receiving only the secondary antibody accompanied each experiment.

### Antibodies

Ms: Mouse; Rb: Rabbit; Gt: Goat.

#### Primary

Ms-Olig2: Millipore MABN50, clone 211F1.1, 1:100 (RRID:AB_10807410). Rb-Olig2: Millipore AB9610, 1:500 (RRID:AB_570666). Rb-Ki67: ThermoFisher, PA5-19462, 1:200 (RRID:AB_10981523). Ms-CC1: Millipore Sigma, OP80, 1:100 (RRID:AB_2057371). Gt-PDGFRα: R&D Systems AF1062, 25 μg, 1:200; 100 μg, 1:60 (RRID:AB_2236897). Gt-PLP: Santa Cruz Biotechnology, G-17, sc-23570 (discontinued), 1:250 (RRID:AB_2165797). Rb-NF200: Sigma-Aldrich N4142, 1:250 (RRID:AB_477272). Ms-Nestin: Millipore Sigma, clone rat-401, MAB353, 1:500 (RRID:AB_94911). Gt-PDGFRβ: R&D Systems AF1042, 1:200 (RRID:AB_2162633). Rb-Laminin: Millipore AB2034, 1:100 (RRID:AB_91209). Rb-NG2: Millipore AB5320, 1:200 RRID:AB_91789). Rb-ALDH1A2: Abcam AB75674, 1:200 (RRID:AB_10676130). Rb-IBA1: Wako Chemicals 019-19741, 1:750 (RRID:AB_839504). Rb-GFAP: Millipore AB5804, 1:500 (RRID:AB_2109645). Rt-CTIP2: Abcam AB18465, 1:500 (RRID:AB_2064130). Rb-TBR1: Abcam AB31940, 1:50 (RRID:AB_2200219). Ms-SATB2: Abcam AB515102, 1:500 (RRID:AB_882455).

#### Secondary

All Alexa Fluor secondaries were raised in donkey and were purchased from ThermoFisher Scientific [anti-mouse 488, R37114 (RRID:AB_2556542); anti-rabbit 488, R37118 (RRID:AB_2556546); anti-goat 488, A-11055 (RRID:AB_2534102); (anti-mouse 594, R37115 (RRID:AB_2556543); anti-rabbit 594 R37119 (RRID:AB_2556547); anti-goat 594, A-11058 (RRID:AB_2534105); and (anti-rabbit 647, A-31573 (RRID:AB_2536183)].

#### Nuclear stain and mounting medium

ProLong Gold Antifade reagent with DAPI (Invitrogen, P36941).

### *In situ* hybridization, RNAscope assay

Reagents were purchased online from Advanced Cell Diagnostics. Slides were removed from the –80°C freezer and immediately fixed in 4% PFA for 15 min at room temperature. Slides were dehydrated through a series of incubations in ethanol at room temperature then subjected to a pretreatment step with the kit’s protease IV and incubated for 30 min at room temperature. Slides were rinsed twice with 1× PBS. The RNAscope assay was performed exactly as described by the manufacturer. Amplification-4C was used for the final step.

#### Probes

*Raldh2*: Mm-Aldh1a2-O1 targeting base pairs 821-2201 of NM_009022.4, catalog #540221-C3. *Olig2*: Mm-Olig2-C2, catalog #447091-C2*. Gli1*: Mm-Gli1-C1, catalog #311001-C1

#### Accessory reagents

Protease III and IV catalog #322340; Multiplex Detection Reagents catalog #320851; Wash Buffer Reagents catalog #310091.

### Terminal deoxynucleotidyl transferase (TdT) dUTP Nick-end labeling (TUNEL) assay

Slides were dried at room temperature for 15 min, fixed in 4% PFA for 30 min at room temperature, then washed twice in 1× TBS for 3 min. Slides were then subjected to the TUNEL assay manufactured by Sigma-Aldrich (Roche, catalog #11684795910) exactly as specified in the manufacturer’s instructions. A positive control slide was treated with DNase I (Sigma-Aldrich D5025; 1 U/10 μl of DNase I buffer, 20 mM Tris-HCl, 2 mM MgCl_2_, and 50 mM KCl; pH 8.4) before application of the TUNEL reaction mixture; a negative control slide received the labeling solution instead of the TUNEL reaction mixture. Slides were then rinsed three times with 1× TBS and mounted with ProLong Gold Antifade reagent with DAPI. Slides were left overnight at room temperature in the dark to dry until imaging.

### Imaging and quantification

#### Immunofluorescence

To ensure that similar regions along the rostral-caudal axis were compared between control and cKO mice, only slides with corresponding numbers were included in a given experiment. For example, in the first experiment, only slide #1 of each animal included in the experiment would be used. When counting cells was not an appropriate measure, we measured the area stained positive for a given marker. To do this, the image was thresholded in Fiji (RRID:SCR_002285; shift + T; left drop down menu: “default”; right drop down menu: “red background”; check “dark background”, adjust slider appropriately so that the red area corresponds to what is judged as positive staining), then the area of the thresholded region was measured (“analyze” tab > set measurements > check “area” and “limit to threshold”; click M). In all cases, to facilitate the analysis, the “polygon selection” tool was used to isolate the CC and the region outside the CC was removed (“edit” tab > clear outside). Cortical thickness was determined using the “straight line” tool in Fiji on DAPI-stained cortical sections imaged with a 4× objective. A line was drawn from the meninges to the bottom of layer 6 and the beginning of the CC, and the length was measured (push “M” and the length will appear in a new pop-up window). The ending point was chosen based on cell density (higher in the layer 6 and lower in the CC) as well as the orientation of cells in the CC (subcallosal white matter cells orient themselves along the axonal tracts coursing across the midline, creating patterns resembling a beaded necklace that is perpendicular to the radial organization of the cortical layers).

#### RNAscope

In RNAscope, fluorescent puncta with diameters of 0.5 μm correspond to single transcripts, while puncta with larger diameters correspond to clusters of transcripts. For *Raldh2* and *Gli1* puncta, the Imaris imaging platform (RRID:SCR_007370) was used to count the number of puncta with diameters ranging from 0.5 to 0.9 μm. Before puncta quantification in Imaris, the images were processed in Fiji. For quantification of *Gli1* puncta, the channel containing *Gli1* staining was masked so that only DAPI was visible. The freehand selection tool was used to trace around the entirety of the SVZ present in the image. The area of the SVZ was recorded in Excel, and the image was cropped, leaving only the SVZ visible. For *Raldh2* puncta, the channel containing *Raldh2* staining was masked, and DAPI staining was used to isolate the midline meninges and the image was cropped. The images of the isolated SVZs and midline meninges were then analyzed using Imaris. Briefly, one image from a given experiment was chosen at random from which to build a dot detection protocol that would then be run in batch and applied to all the images. The settings used to build a dot detection protocol were specific to the probe being used, i.e., the *Gli1* dot detection protocol was different from that used for *Raldh2* owing to differences in signal-to-noise ratios between the different probes.

#### Imaging

Imaging was done on a Nikon Eclipse Ti widefield fluorescence microscope using Nikon Plan Fluor 4× (NA: 0.13), 20× (NA: 0.5), 40× (Oil, NA: 1.3), or 100× (Oil, NA: 1.45) for immunofluorescence. An X-Cite 120 LED (Excelitas Technologies) was used.

## Results

### RALDH2 expression in the postnatal mouse brain

**Figure 2.**
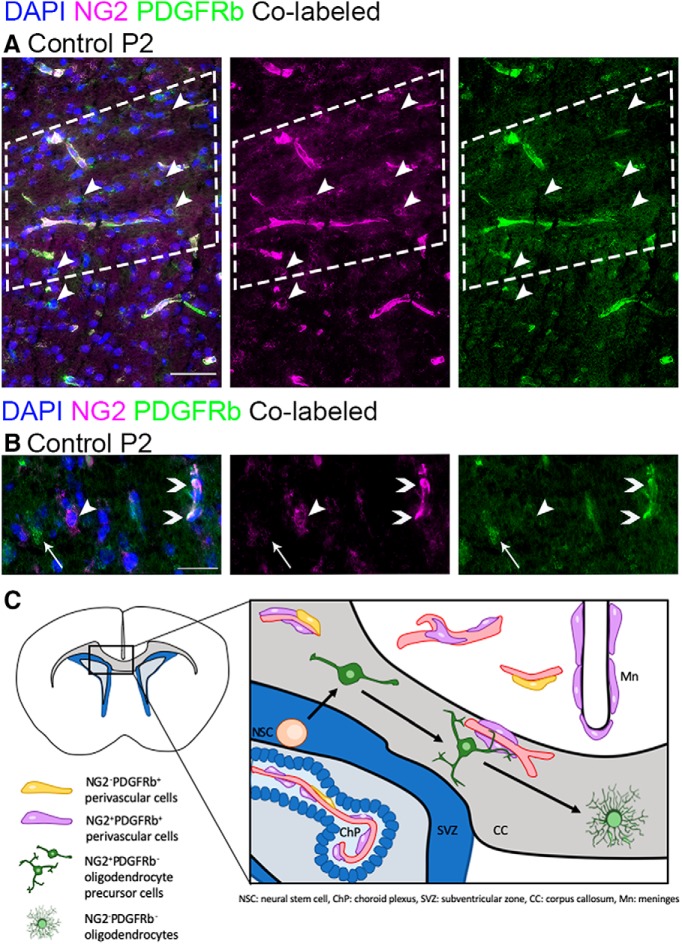
NG2 and PDGFRβ immunofluorescence identifies different perivascular cell populations and OPCs. ***A***, Immunofluorescence for NG2 (magenta) and PDGFRβ (green) in control CC (the area enclosed in the white dashed line) at P2. Co-localization in mural cells is seen in white. NG2^+^PDGFRβ^-^ cells are OPCs (arrowheads). ***B***, In addition to mural cells (white, chevrons) and OPCs (magenta, arrowhead), NG2 and PDGFRβ staining reveals NG2^-^PDGFRβ^+^ cells (green, thin arrow). ***C***, Schematic illustrating the location of these three populations based on our observations. The three black arrows between the NSC and bipolar OPC, the bipolar and multipolar OPC, and the multipolar OPC and highly branched oligodendrocyte represent specification, maturation and differentiation, respectively, in the oligodendrocyte lineage. Scale bars: 50 μm (***A***) and 25 μm (***B***). Mn, meninges; CC, corpus callosum; ChP, choroid plexus; SVZ, subventricular zone; NSC, neural stem cell.

To observe *in vivo* the perivascular cell types relevant to endogenous RALDH2 expression and RA synthesis, we performed immunofluorescence analysis for NG2 and PDGFRβ. NG2 is expressed by OPCs, mural cells (pericytes and SMCs), and some fibroblast-like perivascular cells, type 2 (FB2; Vanlandewijck et al., 2018). The latter two populations are characterized by co-staining with PDGFRβ while OPCs are PDGFRβ^-^. NG2^+^PDGFRβ^-^ OPCs have small, round cell bodies and a halo of NG2 staining around them, the appearance of which is due to the extensive branching of OPC processes (Fig. 2*A*,*B*, arrowheads). NG2^+^PDGFRβ^+^ mural and FB2 cells are elongated in a pattern resembling blood vessels (Fig. 2*A*,*B*, co-staining indicated by white regions in *A* as well as chevrons in *B*). Our staining also revealed NG2^-^PDGFRβ^+^ cells that were elongated like mural and FB2 cells (Fig. 2*B*, thin arrow). This staining pattern likely identifies the FB populations, type 1 and some type 2 cells (FB1 and FB2, respectively; Vanlandewijck et al., 2018). The location of these different cells, based on our analysis of the immunofluorescence images presented here, is depicted in Figure 2*C*.

**Figure 3.**
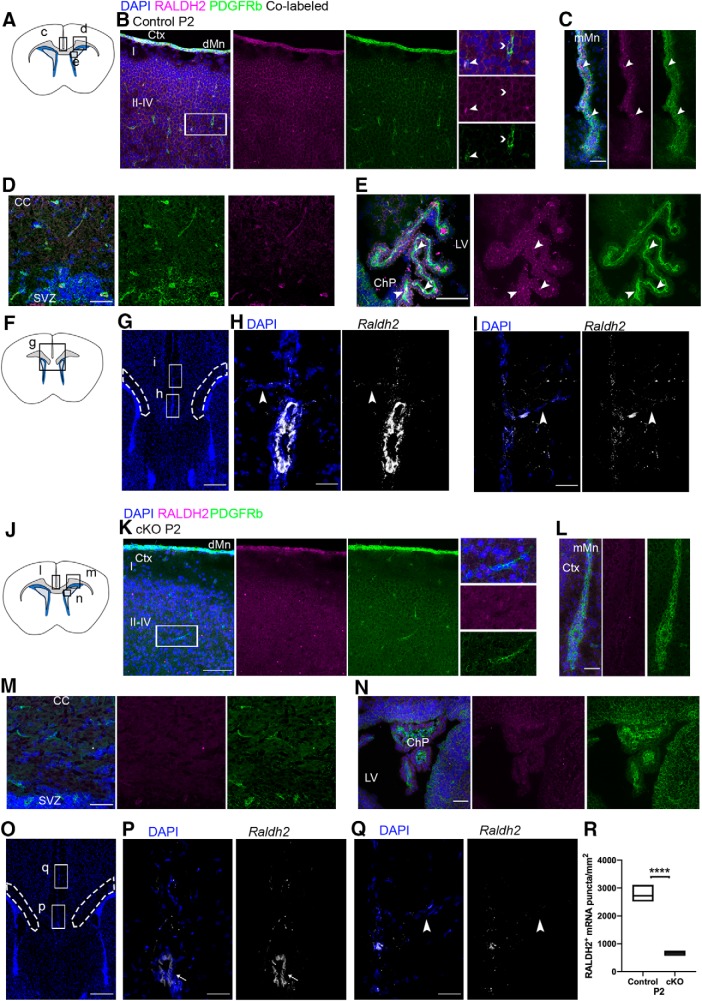
RALDH2 expression in the postnatal forebrain of control and cKO mice. ***A***, Coronal section illustrating regions imaged in panels ***C–E***. ***B***, RALDH2 (magenta) co-localizes with PDGFRβ (green) in the dorsal meninges and blood vessels, with a close up of RALDH2 staining in a PDGFRβ^+^ cell (arrowhead) and a PDGFRβ^+^ cell that does not express RALDH2 (chevron) in the cortex. ***C–E***, RALDH2 co-localizes with PDGFRβ^+^ cells (arrowheads) in the midline meninges (***C***), CC (***D***), choroid plexus stroma (***E***). ***F***, Coronal section illustrating the region imaged in ***G***. ***G***, Image indicating the midline meninges, CC (dotted line), SVZ (dense DAPI below dotted lines), and the regions seen in ***H***, ***I***. ***H***, ***I***, *Raldh2* mRNA puncta are located in the midline meninges, blood vessels entering the brain parenchyma (arrowheads), and a large blood vessel (bright white vertically oriented ellipse in the lower portion of ***H***). ***J***, Coronal section illustrating regions imaged in panels ***L–N***. ***K***, RALDH2 (magenta) is undetectable in the dorsal meninges and parenchymal blood vessels of cKO mice, while PDGFRβ^+^ cells (green) are still present; inset shows close up of a PDGFRβ^+^RALDH2^-^ cell in the cortex. ***L–N***, RALDH2 staining is not present in the midline meninges (***L***), CC (***M***), or choroid plexus (***N***). ***O***, Image indicating the midline meninges, CC, SVZ, and the regions seen in ***P***, ***Q***. ***P***, ***Q***, *Raldh2* mRNA puncta (white) in the meninges, penetrating blood vessels (arrowhead), and a large blood vessel (thin arrow) are reduced by 76% compared to controls. ***R***, Quantification of *Raldh2* mRNA puncta per millimeter squared. Ctx, cortex; dMn, dorsal meninges; CC, corpus callosum; ChP, choroid plexus; SVZ, subventricular zone; LV, lateral ventricle; mMn, midline meninges; cKO, conditional knock-out. Scale bars: 200 μm (***G***, ***O***), 100 μm (***B***, ***C***, ***E***, ***K***, ***L***, ***N***), 25 μm (***D***, ***M***), and 10 μm (***H***, ***I***, ***P***, ***Q***). Floating bar plot, box: minimum to maximum value, line at median. ****p < 0.0001.

To characterize RALDH2 expression in the postnatal CNS, immunofluorescence analysis for RALDH2 in the brains of wildtype mice at P2 was performed with PDGFRβ. We found that RALDH2^+^ cells co-localized with PDGFRβ^+^ (Fig. 3*B*, right insets, arrowhead), but not all PDGFRβ^+^ cells were RALDH2^+^ (Fig. 3*B*, right insets, chevron). Due to primary antibody incompatibilities, we were unable to perform RALDH2 co-staining with NG2 or Col1a1. Our results suggest that RALDH2 is expressed in PDGFRβ^+^ cells in the meninges, along parenchymal blood vessels in the cortex and subcortical white matter, and choroid plexus stroma (Fig. 3*B–E*).

Since the meninges, choroid plexus, and vasculature are vulnerable to autofluorescence, we performed RNAscope *in situ* hybridization to confirm the location of *Raldh2* expression in the CNS (Fig. 3*G–I*). We found that *Raldh2* mRNA was only detectable in the meninges and in blood vessels immediately adjacent to the meninges (Fig. 3*H*,*I*). We did not detect *Raldh2* mRNA in other blood vessels deeper in the gray and white matter despite the presence of RALDH2 immunostaining in these structures. *Raldh2* mRNA staining was most clearly visible in the midline meninges. This region is more frequently left intact after dissection of the brain from the skull, which can damage or unintentionally remove the dorsal meninges. Furthermore, this region is the closest meningeal tissue to regions relevant to OL lineage cell development such as the subcortical white matter and the subcallosal SVZ. We did not detect RALDH2 protein or mRNA in OPCs or other CNS cells at the time points analyzed. These results suggest that RALDH2 is mainly expressed in a subset of PDGFRβ^+^ cells in the postnatal brain. Moreover, several studies have observed RALDH2 expression in NG2+ glia and mature OLs following spinal cord injury (Mey et al., 2005; Kern et al., 2007; Goncalves et al., 2018), thus implicating RALDH2-dependent RA synthesis in NG2^+^ cells in OPC differentiation following injury. However, our examination of expression profiles databases generated by recent single cell RNA sequencing studies aimed at characterizing OL lineage cell progression and heterogeneity in the adult CNS revealed that *Raldh2* was not expressed highly by any cell in the OL lineage (Marques et al., 2016, 2018). Nevertheless, all these findings suggest considerable overlap between RALDH2 and NG2 expression in the postnatal CNS and raise the possibility that in some cases OL lineage cells express RALDH2.

**Figure 4.**
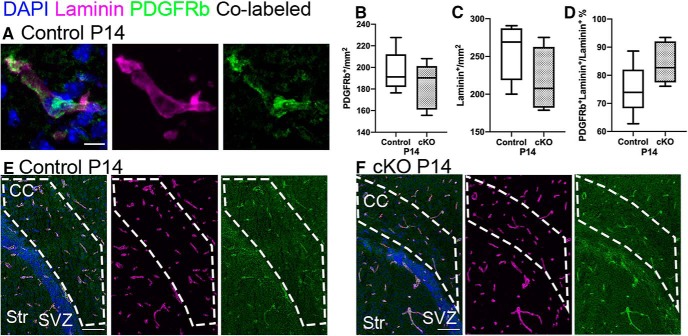
PDGFRβ^+^ cells and vasculature are normal in the *Raldh2* cKO. ***A***, PDGFRβ^+^ cells (green) co-localize with Laminin on blood vessels. ***B***, Quantification of PDGFRβ^+^ structures. ***C***, Quantification of Laminin^+^ structures. ***D***, Percentage of Laminin^+^ structures co-localizing with PDGFRβ. ***E***, ***F***, Immunofluorescence for Laminin (magenta) and PDGFRβ (green), in the CC (area within the white dashed line) of control and cKO mice; co-localization shown in white. Mn, meninges; Ctx, cortex; CC, corpus callosum; SVZ, subventricular zone; LV, lateral ventricle; ChP, choroid plexus; Str, striatum; cKO, conditional knock-out. Scale bars: 50 μm (***E***, ***F***) and 5 μm (***A***). Box and whiskers plot, box: 25th and 75th percentile, whiskers: minimum to maximum value, line at median.

In light of this, we generated an *Ng2-Cre:Raldh2^flox/flox^* mouse line, hereafter referred to as the *Raldh2* cKO. We found that *Raldh2* cKO mice were born normally and did not display any gross abnormalities. The absence of RALDH2 in PDGFRβ^+^ cells in the meninges, choroid plexus stroma, and parenchymal blood vessels was confirmed in the *Raldh2* cKO by immunofluorescence (Fig. 3*K–N*). Moreover, RNAscope analysis revealed that the expression of *Raldh2* in the midline meninges was decreased by 76% in cKO mice, consistent with the previously reported 80% efficacy of Cre recombinase in the *Ng2-Cre* line (Zhu et al., 2008; Fig. 3*O*–*R*; *n* is number of animals, *n* = 3 controls, *n* = 4 cKOs, unpaired *t* test between genotypes at P2, one-tailed, *t*_(5)_ = 13.85, *p* < 0.0001^a^). To determine whether *Raldh2* cKO affected the number of blood vessels or PDGFRβ^+^ cells, which could indirectly impact OL development, we performed immunofluorescence with PDGFRβ and the basal lamina marker Laminin to mark vasculature at P14, during peak OPC production and maturation (Fig. 4*A*). We show that the number of PDGFRβ^+^ cells, the blood vessels, and blood vessel coverage by PDGFRβ^+^ cells were not altered in the absence of RALDH2 (Fig. 4*B–F*, quantification of PDGFRβ^+^ structures: *n* is number of animals, *n* = 5/genotype, unpaired *t* test between genotypes at P14, two-tailed, *t*_(8)_ = 1.001, *p* = 0.3463^b^; quantification of Laminin^+^ structures: *n* is number of animals, *n* = 5/genotype, unpaired *t* test between genotypes at P14, two-tailed, *t*_(8)_ = 1.462, *p* = 0.1820^c^; percentage of Laminin^+^ structures co-localizing with PDGFRβ, *n* is number of animals, *n* = 5/genotype, unpaired *t* test between genotypes at P14, two-tailed, *t*_(8)_ = 1.784, *p* = 0.1122^d^).

### Loss of RALDH2 reduces OPC and OL numbers

**Figure 5.**
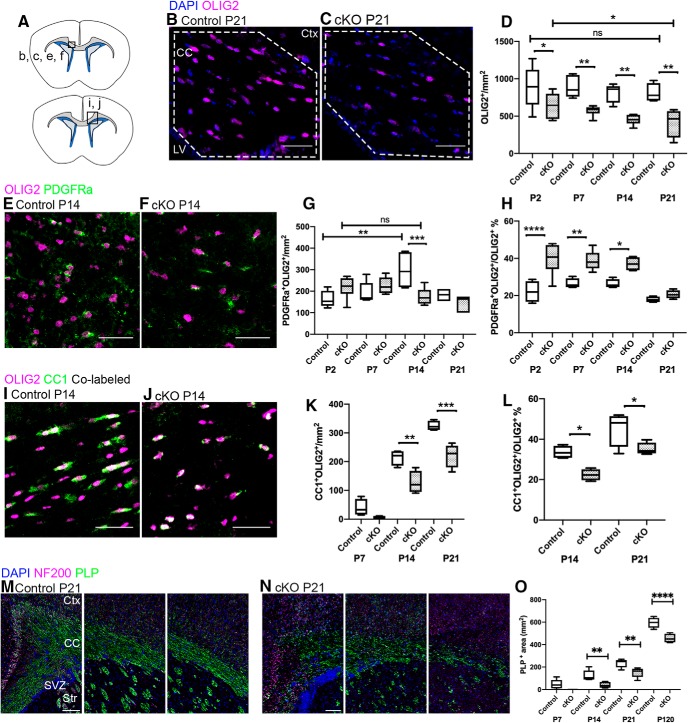
*Raldh2* cKO results in reduced numbers of OPCs and OLs. ***A***, Coronal section illustrating the regions imaged in ***B***, ***C***, ***E***, ***F***, and ***M***, ***N*** (top) and ***I***, ***J*** (bottom). ***B***, Control mice have abundant OL lineage cells (OLIG2, magenta; CC, dotted line) and OLIG2^+^ cell numbers remain stable throughout the first three weeks of postnatal life. ***C***, cKO mice have significantly fewer OLIG2^+^ cells at all time points examined. ***D***, Quantification of OLIG2^+^ cell number in control versus cKO mice at each time point. ***E***, ***F***, Control and cKO mice both have OPCs [co-labeled by PDGFRα (green) and OLIG2 (magenta)]. ***G***, control and cKO mice have comparable total PDGFRα^+^OLIG2^+^ cell numbers at all time points except P14, when cKO mice have fewer, and while controls displayed at least a two-fold increase in OPCs between P2 and P14, the pool of OPCs in cKO mice did not expand over the same time period. ***H***, More OLIG2^+^ cells are co-labeled with PDGFRα in cKO mice than controls. ***I***, ***J***, Control and cKO mice both have mature OLs [co-labeled by CC1 (green) and OLIG2 (magenta)]. ***K***, cKO mice have reduced CC1^+^OLIG2^+^ cell numbers at P14 and P21. ***L***, In cKO mice, fewer OLIG2^+^ cells are co-labeled with CC1 than in controls. ***M***, ***N***, Control mice have more PLP^+^ labeled area (green) than cKO mice at P14, P21, and P120; NF200 (magenta) labels axons. ***O***, Quantification of reduced PLP labeling. CC, corpus callosum; Ctx, cortex; LV, lateral ventricle; SVZ, subventricular zone; Str, striatum; cKO, conditional knock-out. Scale bars: 100 μm (***M***, ***N***) and 50 μm (***B***, ***C***, ***E***, ***F***, ***I***, ***J***). Box and whisker plot, box: 25th and 75th percentile, whiskers: minimum to maximum value, line at median. Floating bar plot, box: minimum to maximum value, line at median. *p < 0.05, **p < 0.01, ***p < 0.001, ****p < 0.0001. ns = not significant.

To determine whether RA synthesis by *Ng2-Cre*-recombined cells is required for normal OL development *in vivo*, we quantified the number of OL lineage cells, comprising OPCs and OLs, using the lineage marker OL transcription factor 2 (OLIG2; Zhou et al., 2000; Zhou and Anderson, 2002). OL lineage cells were analyzed in the CC at four time points that correspond to developmental OL lineage cell progression (P2, P7, P14, and P21). We observed significantly fewer OLIG2^+^ cells in cKO mice than controls at all time points examined (Fig. 5*B–D*; *n* is number of animals, *n* = 4–6/genotype, one-way ANOVA with Sidak’s test for multiple comparisons between genotypes at individual time points P2–P21, *F*_(7,35)_ = 7.933, *p* < 0.0001^e^). In controls, total OLIG2^+^ cell numbers did not significantly change over the first three weeks of life (Fig. 5*D*; *n* is number of animals, *n* = 5 P2 controls, *n* = 4 P21 controls, unpaired *t* test within genotype between individual time points P2 vs P21, one-tailed, *t*_(7)_ = 0.5192, *p* = 0.3098^f^), while in cKO, OLIG2^+^ cell numbers were reduced between P2 and P21 (Fig. 5*D*; *n* is number of animals, *n* = 6 P2 cKOs, *n* = 4 P21 cKOs, unpaired *t* test within genotype between individual time points P2 vs P21, one-tailed, *t*_(8)_ = 1.962, *p* = 0.0427^g^).

To determine whether the reduction of OLIG2^+^ cells in the cKO resulted from a decrease in OPCs or OLs, or both, the number of OPCs and OLs in cKO and control mice in the CC was quantified at P2, P7, P14, and P21 via immunofluorescence. Quantification of OPC numbers, based on co-expression of OLIG2 and PDGFRα, a marker of OPCs (Fig. 5*E*,*F*), revealed that cKO and control mice had comparable PDGFRα^+^ OPC numbers at all time points except P14, when cKO OPC numbers were decreased by >40% relative to controls (Fig. 5*G*; *n* is number of animals, *n* = 6/genotype, one-way ANOVA with Sidak’s test for multiple comparisons between genotypes at individual time points P2–P21, *F*_(7,34)_ = 4.814, *p* = 0.0008^h^). Moreover, while controls displayed at least a two-fold increase in OPCs between P2 and P14 (Fig. 5*G*; *n* is number of animals, *n* = 3–7 controls/time point, one-way ANOVA with Sidak’s test for multiple comparisons within genotype between individual time points P2–P21, *F*_(3,7)_ = 6.728, *p* = 0.0034^i^), the pool of OPCs in cKO mice did not expand over the same time period. However, we found that PDGFRα^+^ OPCs constituted a significantly larger percentage of the OLIG2^+^ cell population in cKO mice than in controls at all time points (Fig. 5*H*; *n* is number of animals, *n* = 4–6/genotype, one-way ANOVA with Sidak’s test for multiple comparisons between genotypes at individual time points P2–P21, *F*_(7,27)_ = 13.98, *p* < 0.0001^j^), suggesting that OL differentiation might be affected. To examine OLs, co-immunostaining analysis of OLIG2 and CC1, which is involved in mature OL production (Lang et al., 2013), was performed at P7, P14, and P21 (Fig. 5*I*,*J*). We found that the number of CC1^+^ OLs in the CC progressively increased from P7 to P21 in both the cKO and control. However, the cKO displayed fewer CC1^+^ OLs compared to controls at P14 and P21 (Fig. 5*K*; *n* is number of animals, *n* = 4/genotype, one-way ANOVA with Sidak’s test for multiple comparisons between genotypes at individual time points P7–P21, *F*_(5,18)_ = 68.26, *p* < 0.0001^k^). Correspondingly, the percentage of CC1^+^ OLs was significantly reduced among the total OLIG2^+^ pool in cKO mice compared to control littermates (Fig. 5*L*; *n* is number of animals, *n* = 4/genotype, one-way ANOVA with Sidak’s test for multiple comparisons between genotypes at individual time points P14–P21, *F*_(3,12)_ = 13.96, *p* = 0.0003^l^). To confirm the OL deficit, we quantified the expression of proteolipid protein (PLP), an integral myelin protein, in the CC of cKO and control mice. We found that PLP^+^ labeling in the cKO was reduced at P14 and P21, contrasting sharply with its high expression in control brains at the same time points (Fig. 5*M–O*; *n* is number of animals, *n* = 5–6/genotype, one-way ANOVA with Sidak’s test for multiple comparisons between genotypes at individual time points P7–P120, *F*_(7,32)_ = 134.2, *p* < 0.0001^m^). We also found that this reduction in PLP persisted at four months (P120), suggesting this phenotype is a deficit in OL development rather than a delay. Together, our results suggest that endogenous RALDH2 supports the differentiation of OPCs into OLs.

### Loss of RALDH2 reduces NSC survival and *Gli1* expression in the SVZ but does not affect proliferation or survival of OL lineage cells

**Figure 6.**
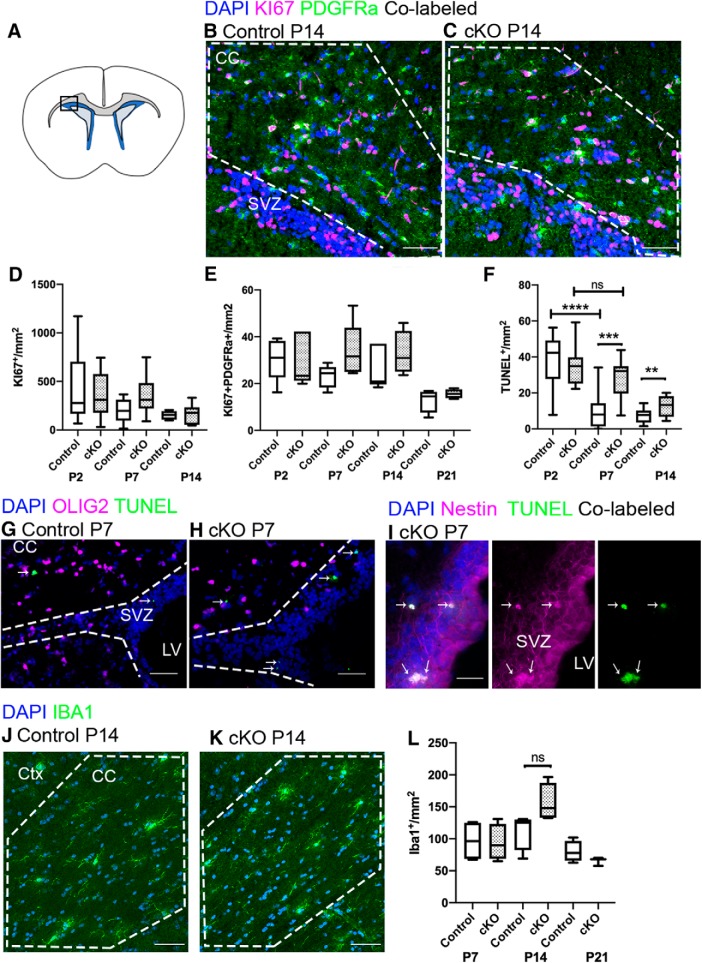
NSC survival is reduced in the *Raldh2* cKO. ***A***, Coronal section illustrating the regions imaged in the other panels. ***B***, ***C***, Control and cKO mice both have proliferating PDGFRα^+^ cells (green) identified by co-labeling (white) with KI67 (magenta), and non-proliferating PDGFRα^+^ cells. ***D***, ***E***, Control and cKO mice have comparable numbers of total KI67^+^ cells and KI67^+^PDGFRα^+^ cells at all time points. ***F***, Between P2 and P7 in control animals, the number of TUNEL^+^ cells dropped significantly. Between P2 and P7 in cKO mice, TUNEL^+^ cell numbers were unchanged. At P7 and P14, cKO mice had significantly more TUNEL^+^ cells than controls. ***G***, ***H***, Dying cells identified by labeling with TUNEL (green, thin arrow) were present in the CC and SVZ (delineated by dotted lines) of both controls and cKO, though no OLIG2^+^ cells were co-labeled with TUNEL. ***I***, In the cKO SVZ, 76% of all TUNEL^+^ cells were co-labeled by Nestin [magenta, co-labeled cells are white (thin arrow)]. ***J***, ***K***, Immunofluorescence for IBA1^+^ in the CC of control and cKO mice. ***L***, Quantification of the number of IBA1^+^ cells shows no significant difference between groups; a transient increase in IBA1^+^ cells appears at P7 in cKO mice but does not reach significance. CC, corpus callosum; SVZ, subventricular zone; LV, lateral ventricle; cKO, conditional knock-out. Scale bars: 50 μm (***B***, ***C***, ***G***, ***H***, ***J***, ***K***) and 25 μm (***I***). Box and whisker plot, box: 25th and 75th percentile, whiskers: minimum to maximum value, line at median. **p < 0.01, ***p < 0.001, ****p < 0.0001. ns = not significant.

Reduced OPC numbers in the CC of two-week-old cKO mice could be due to decreased OPC proliferation or increased OPC death. To determine whether RALDH2 affects OPC proliferation, immunofluorescence analysis of PDGFRα and KI67 co-labeling in the CC was performed at P2, P7, P14, and P21 (Fig. 6*B*,*C*). We found the total numbers of KI67^+^ cells were not significantly different between cKO and controls at any time point examined (Fig. 6*D*). Moreover, the number of proliferating OPCs between cKO and controls were similar to each other across all time points (Fig. 6*E*). To determine whether increased cell death played a role in the overall OLIG2^+^ cell deficit in the cKO mice, the TUNEL assay was performed to detect cleaved DNA in dying cells at P2, P7, and P14. There was a significant reduction of TUNEL^+^ cells between P2 and P7 in the CC and SVZ of control mice (Fig. 6*F*; *n* is number of animals, *n* = 8 P2 controls, *n* = 10 P7 controls, unpaired *t* test within genotype between individual time points P2 vs P7, one-tailed, *t*_(16)_ = 4.727, *p* < 0.0001^n^). This reduction was not present in cKO mice between P2 and P7 (Fig. 6*F*; *n* is number of animals, *n* = 8 P2 cKOs, *n* = 10 P7 cKOs, unpaired *t* test within genotype between individual time points P2 vs P7, one-tailed, *t*_(16)_ = 1.222, *p* = 0.1197^°^). At P2, control and cKO mice had similar levels of TUNEL^+^ cells, however, the number of TUNEL^+^ cells in the cKO was significantly increased at P7 and P14 compared to control littermates (Fig. 6*F*; *n* is number of animals, *n* = 10/genotype, one-way ANOVA with Sidak’s test for multiple comparisons between genotypes at individual time points P2–P14, *F*_(5,50)_ = 16.24, *p* < 0.0001^p^). Interestingly, we did not find TUNEL co-labeling with OLIG2 in either control or cKO mice (Fig. 6*G*,*H*). Instead, we found that the majority of TUNEL^+^ cells in the cKO mice was detected at the SVZ and co-labeled with the NSC marker, Nestin (Fig. 6*I*). These results suggested that *Raldh2* cKO did not affect OPC proliferation or survival, but impaired NSC survival in the SVZ.

We also examined whether loss of RALDH2 expression affected microglial development. Microglial activation, including increased proliferation, can be mitigated by RA signaling (Dheen et al., 2005; Takamura et al., 2017), and changes in microglial function could impact OL development. By performing immunofluorescence staining for ionized calcium-binding adaptor molecule 1 (IBA1) at P7, 14, and P21, we found no significant differences between genotypes in terms of IBA1^+^ cell number (Fig. 6*J–L*; *n* is number of animals, *n* = 3–4/genotype, one-way ANOVA with Sidak’s test for multiple comparisons between genotypes at individual time points P7–P21, *F*_(5,17)_ = 1.028, *p* = 0.4326^q^), suggesting that loss of RALDH2 does not impact OL lineage cell development through changes in microglial cell number.

**Figure 7.**
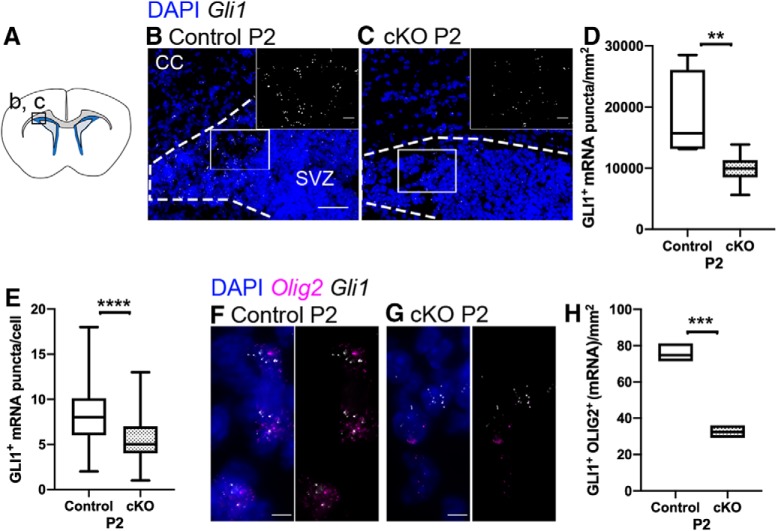
*Gli1* expression is reduced in the SVZ in *Raldh2* cKO mice. ***A***, Coronal section illustrating the region imaged in ***B***, ***C***. ***B***, ***C***, In controls and cKO mice, *Gli1* mRNA puncta (white) are found in the SVZ (delineated by dotted lines) and the transition from the SVZ into the CC (area flanking the top dotted line in ***B***, ***C***). ***D***, The total number of *Gli1* puncta in the SVZ and its border with the CC is reduced in cKO at P2 relative to controls. ***E***, The number of *Gli1* mRNA puncta per cell is reduced in cKO mice. ***F***, ***G***, *Gli1*^+^*Olig2*^+^-co-labeled cells (white and magenta) cells are found at the border between the SVZ and CC in controls and cKO. ***H***, The number of *Gli1*^+^*Olig2*^+^ cells is reduced in cKO mice. CC, corpus callosum; SVZ, subventricular zone; LV, lateral ventricle; cKO, conditional knock-out. Scale bars: 50 μm (***B***, ***C***) and 5 μm (***F***, ***G***). Box and whisker plot, box: 25th and 75th percentile, whiskers: minimum to maximum value, line at median. Floating bar plot, box: minimum to maximum value, line at median. **p < 0.01, ***p < 0.001, ****p < 0.0001.

The persistent cell death among NSCs in *Raldh2* cKO mice between P2 and P14 led us to ask whether OPC production from NSCs in the SVZ was affected in the absence of RALDH2. It has previously been demonstrated that transient SHH signaling in NSCs in the SVZ contributes to the generation of the majority of OPCs in the postnatal CC (Tong et al., 2015; Traiffort et al., 2016; Sanchez and Armstrong, 2018; Winkler et al., 2018). To determine whether decreased SHH signaling contributed to OL lineage cell deficits in *Raldh2* cKO mice, we performed RNAscope *in situ* hybridization analysis for the expression of *Gli1*, an indicator of active SHH signaling, and *Olig2* (Fig. 7*B*,*C*,*F*,*G*). We found that cKO mice displayed an almost 50% reduction of *Gli1* transcripts in the SVZ compared to controls at P2 (Fig. 7*D*; *n* is number of animals, *n* = 5 controls, *n* = 7 cKOs, unpaired *t* test between genotypes at P2, one-tailed, *t*_(10)_ = 3.251, *p* = 0.0044^r^), and the number of *Gli1* ^+^ puncta per cell was also reduced (Fig. 7*E*; *n* is number of cells included in the analysis of three mice per genotype, *n* = 66 in controls, *n* = 80 in cKOs, unpaired *t* test between genotypes at P2, one-tailed, *t*_(144)_ = 5.363, *p* < 0.0001^s^). At the transition between the CC and subcallosal SVZ, we detected significantly fewer *Gli1* ^+^*Olig2* ^+^ cells in cKO mice than controls (Fig. 7*F–H*; *n* is number of animals, *n* = 3/genotypes, unpaired *t* test between genotypes at P2, one-tailed, *t*_(4)_ = 12.36, *p* = 0.0001^t^). These results suggest that loss of RALDH2 expression could have affected SHH-mediated production of subcortical white matter OPCs from the subcallosal SVZ.

### Loss of RALDH2 reduces GFAP labeling in the CC and alters cortical neuron numbers

**Figure 8.**
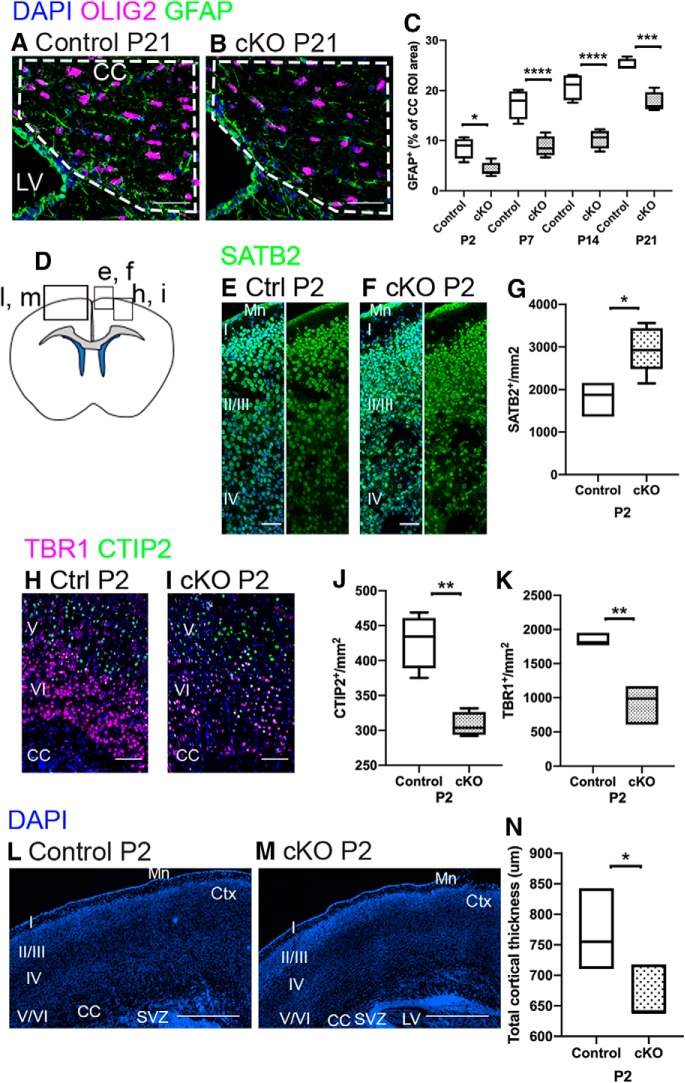
Other NSC-derived cell types are reduced by loss of RALDH2. ***A***, ***B***, Control and cKO mice both have GFAP^+^ expression in the CC (dotted lines). OLIG2 marks OL lineage cells and is included to provide contrast. ***C***, The area of CC that is labeled by GFAP is significantly reduced in cKO mice relative to controls at all time points examined. ***D***, Coronal section illustrating the regions imaged in ***E***, ***F***, ***H***, ***I***, ***L***, ***M***. ***E–G***, cKO mice have significantly more SATB2^+^ neurons (green) than controls. ***H***, ***I***, cKO mice have significantly fewer layer 6 (TBR1, magenta) and layer 5 (CTIP2, green) neurons than control mice at P2. ***J***, Quantification of CTIP2^+^ cell reduction in cKO mice. ***K***, Quantification of TBR1^+^ cell reduction in cKO mice. ***L***, ***M***, Cortical thickness is reduced in cKO mice. ***N***, Quantification of cortical thickness. CC, corpus callosum; LV, lateral ventricle; Ctx, cortex (with layers 1–6 in roman numerals); SVZ, subventricular zone; Mn, meninges; cKO, conditional knock-out. Scale bars: 200 μm (***L***, ***M***), 100 μm (***A***, ***B***), 50 μm (***H***, ***I***), and 25 μm (***E***, ***F***). Box and whisker plot, box: 25th and 75th percentile, whiskers: minimum to maximum value, line at median. Floating bar plot, box: minimum to maximum value, line at median. *p < 0.05, **p < 0.01, ***p < 0.001, ****p < 0.0001.

The possibility that loss of *Raldh2* affects OL lineage cell numbers via altered NSC function led us to ask whether other NSC-derived cell populations, specifically cortical neurons and astrocytes, were also impacted. First, we asked if astrocytes in the CC were affected in the *Raldh2* cKO mice by performing glial fibrillary acidic protein (GFAP) immunostaining. We found that cKO mice exhibited a two-fold decrease in GFAP^+^ area at all time points examined (Fig. 8*A–C*; *n* is number of animals, *n* = 4–6/genotype, one-way ANOVA with Sidak’s test for multiple comparisons between genotypes at individual time points P2–P21, *F*_(7,25)_ = 52.95, *p* < 0.0001^u^). These findings suggest that astrocyte development is altered in the absence of RALDH2.

Next, to examine cortical neurons, we counted the number of late-born, upper layer neurons based on SATB2 staining. We found a significant increase in SATB2^+^ neuron numbers in cKO mice at P2 relative to controls (Fig. 8*E–G*; *n* is number of animals, *n* = 3–5/genotype, unpaired *t* test between genotypes at P2, one-tailed, *t*_(6)_ = 2.874, *p* = 0.0141^v^). Moreover, SATB2^+^ nuclei in cKO mice seemed smaller than those in controls. Next, we counted the number of early-born, deep layer neurons based on CTIP2^+^ staining of layer 5 neurons and TBR1^+^ staining of layer 6 neurons in control and cKO mice at P2. We found that TBR1^+^ and CTIP2^+^ deep layer neurons were significantly reduced in cKO mice relative to control littermates (Fig. 8*H–K*; quantification of CTIP2^+^ cells: *n* is number of animals, *n* = 4/genotype, unpaired *t* test between genotypes at P2, two-tailed, *t*_(6)_ = 5.631, *p* = 0.0013^w^; quantification of TBR1^+^ cells: *n* is number of animals, *n* = 3/genotype, unpaired *t* test between genotypes at P2, two-tailed, *t*_(4)_ = 5.298, *p* = 0.0061^x^). Gross forebrain morphology was unaffected and cortical layers were clearly discernable by DAPI staining, but cortical thickness in *Raldh2* cKO mice was reduced relative to controls at P2 (Fig. 8*L–N*; *n* is the number of animals, *n* = 3/genotype, unpaired *t* test between genotypes at P2, one-tailed, *t*_(4)_ = 2.20, *p* = 0.0453^y^). As was observed for SATB2^+^ upper layer neurons, we found that nuclei of deep layer neurons appeared smaller compared to control mice. The apparent reduction in nucleus size (and presumably cell soma size) among cortical neurons, as well as the visible decrease in space between the neurons may explain the reduced cortical thickness in cKO mice. This is reminiscent of cell size scaling, which has been observed as a method to avoid increasing brain size in the case of increased neuron number (Herculano-Houzel et al., 2006).

Overall, our results suggest that endogenous RALDH2 influences not only the production and maturation of OPCs in the CC, but also the development cortical neurons and callosal astrocytes.

## Discussion

### RALDH2 expression in the postnatal mouse forebrain

Our goal was to determine whether OL development requires RALDH2. First, we confirmed that postnatal RALDH2 expression overlaps with that of PDGFRβ in the meninges, along gray and white matter blood vessels, and in the choroid plexus, all of which is consistent with previous literature (Yamamoto et al., 1996; Niederreither et al., 1997; Smith et al., 2001; Wagner et al., 2002; Kelly et al., 2016; Vanlandewijck et al., 2018). Additionally, several papers have shown that RALDH2 is found in isolated hippocampal and hindbrain neurons, but we did not observe RALDH2 expression in forebrain neurons (Smith et al., 2001; Wagner et al., 2002). Not all of these studies, however, found the same spatiotemporal expression profiles of RALDH2.

These incongruities may result from differences in methodologies, but some may be physiologically relevant and inherent to RALDH2. For example, RALDH2 protein expression patterns are variable within samples and within time points in the olfactory neuroepithelium where retinoids influence proliferation, differentiation, and survival of olfactory sensory neurons (Asson-Batres et al., 2003; Asson-Batres and Smith, 2006; Hägglund et al., 2006). In fact, several studies propose that there is discontinuity between *Raldh2* mRNA and protein expression *in vivo* (Niederreither et al., 1997; Norlin et al., 2001; Asson-Batres and Smith, 2006).These previous findings may explain why, in control animals, we found abundant *Raldh2* transcripts in the meninges but none in the brain parenchyma, despite RALDH2 protein being visible in perivascular cells.

### RALDH2^+^ cell identity and knock-out of RALDH2 in *Ng2-Cre* lineage cells

This inherent difficulty of RALDH2 detection complicates the task of characterizing RALDH2^+^ cells in the postnatal forebrain. Several studies describe RALDH2 expression in NG2^+^ cells in adult spinal cord parenchyma, ostensibly pericytes (Mey et al., 2005; Kern et al., 2007), but others show that RALDH2 protein and mRNA are found primarily in Col1a1^+^PDGFRβ^+^ cells in a “fibroblast-like” population of perivascular cells (FB cells) and not *Ng2^+^* pericytes (Kelly et al., 2016; Vanlandewijck et al., 2018). There are two FB subpopulations (FB1 and FB2) that differ largely in terms of their collagen expression patterns, and *Raldh2* and *Ng2* are found in varying amounts in both subgroups. Here, through NG2 and PDGFRβ staining, we identified NG2^+^PDGFRβ^-^ cells (OPCs), NG2^+^PDGFRβ^+^ cells (mural cells, consisting of pericytes and SMCs, and likely some FB2 cells), and NG2^-^PDGFRβ^+^ cells (likely FB1 and 2 cells). We found that RALDH2^+^ cells were always PDGFRβ^+^ while not all PDGFRβ^+^ cells were RALDH2^+^. This is consistent with our interrogation of the data in the online gene expression database generated by Vanlandewijck et al. (2018), leading to the conclusion that only a subset of *Pdgfrβ^+^* perivascular cells (i.e., FB cells) express *Raldh2*.

*Raldh2* excision in FB cells of the *Ng2-Cre:Raldh2^flox/flox^* mouse is likely dependent on their expression of NG2 early in development. Several single cell RNA sequencing studies discovered an *Ng2^+^Col1a1^+^* population present in the embryonic mouse brain (Marques et al., 2016, 2018), likely the embryonic precursors of postnatal Col1a1^+^RALDH2^+^ FB cells. While *Ng2* expression is low in postnatal FBs, even transient expression and Cre activation during embryonic development could be enough to affect their expression of *Raldh2* later on. For example, transient *Ng2* expression in NSCs is sufficient to activate the reporter in the astrocytic progeny of the *Ng2-CreBAC:Z/EG* line (Zhu et al., 2008). The Col1a1^+^RALDH2^+^ population in the forebrain expands in the first three postnatal weeks (Kelly et al., 2016). This is also at peak OPC production from the SVZ (P0–P14) and a time when the first mature OLs appear in the CC (P8; Foran and Peterson, 1992; Dai et al., 2015). This may constitute an important time and place for interactions between RALDH2^+^ perivascular cells, NSCs, and OPCs that could influence OLC development.

### RALDH2 amid a multiplicity of RALDHs

RALDH1–RALDH3 are all expressed in forebrain structures, and while each enzyme possesses its own unique spatiotemporal expression profile, a given enzyme’s timing or region of expression may be very close to that of another RALDH isozyme. Importantly, RALDH3 expression in the superior layers of the cortex at P1.5 is found in bipolar, early postmigratory neurons, and a few days later at P5, it is present during arborization of these neurons, suggesting a role in cortical neuron maturation and plasticity (Wagner et al., 2006).

Despite their structural and functional similarities, as well as their proximity to each other in space and time, there is little evidence for redundancy among RALDHs. *Raldh2* null mice die at E9.5 due to failed development of many critical tissues, including the rostral CNS (Kumar et al., 2012), but *Raldh3* expression in the anterior CNS and craniofacial regions is not different between control and *Raldh2* null mice, indicating no compensatory increase in *Raldh3*. Loss of *Raldh2* at E10.5 is sufficient to alter the migratory behavior, laminar position, and molecular identity of some cortical neurons (Haushalter et al., 2017), suggesting that, here again, RALDH3 does not compensate, despite its presence within the cortical layers (Smith et al., 2001).

An explanation for this lack of redundancy could be that each enzyme or combination of enzymes serves a unique purpose, such as providing protection from toxicity through rapid clearance of excess retinoids or producing RA for signaling. The diverse phenotypes found in mouse mutants of retinoid synthesis, degradation, and signaling components suggest that this is the case (Kam et al., 2012). Moreover, it has been hypothesized that the pool of RA generated by a given RALDH enzyme functions differently than that of another RALDH isozyme. A similar phenomenon involving enzymes regulating fatty acid metabolism has been observed in primary astrocyte cultures (Wakil and Abu-Elheiga, 2009; Napoli, 2012).

### RALDH2 is required for OPC production and differentiation

Our results show that OLC numbers decrease following RALDH2 cKO. While controls displayed significant OPC population expansion between P2 and P14, the pool of OPCs in cKO mice did not grow over the same period, and OPC density was decreased by >40% compared to controls. However, we found that these events were not attributable to impaired OPC proliferation or increased OLC death. Instead, they may be the result of insufficient generation of OPCs from the SVZ. This could be related to the increased death of Nestin^+^ cells in the *Raldh2* cKO SVZ, a finding that aligns with reports that RA is necessary for NSC survival (Crandall et al., 2004; Jacobs et al., 2006; Rajaii et al., 2008; Harrison-Uy et al., 2013; Mishra et al., 2016; Wu et al., 2016). The reduction in OPCs may also follow from reduced *Gli1*, indicative of decreased SHH signaling, in the subcallosal SVZ, where SHH is required for OPC production during early postnatal life (Tong et al., 2015; Sanchez and Armstrong, 2018; Winkler et al., 2018). In fact, it has been shown that loss of SHH signaling in postnatal Nestin^+^ cells increased cell death in the SVZ, decreased SHH-dependent production of OPCs, and lead to a subsequent 30% reduction in callosal MBP expression (Machold et al., 2003).

SHH signaling is important for OPC specification in part because of its influence on *Olig2*, and there is evidence that RA is necessary for SHH-dependent *Olig2* expression either by directly enhancing *Shh* transcription via an upstream RARE or regulating SHH target gene expression, in particular that of *Olig2* (Chang et al., 1997; Briscoe and Ericson, 1999; Ribes et al., 2006; Tan et al., 2006; Calder et al., 2015). A similar phenomenon may exist in dorsal forebrain OPC progenitors and may be disrupted by the loss of RALDH2.

Our findings also indicate that RALDH2 regulates the maturation of OPCs. cKO mice displayed a deficit in CC1^+^ OLs relative to the total OLIG2^+^ population, suggesting that OPCs fail to differentiate. RA has been shown to regulate OPC maturation *in vitro* by favoring differentiation over continued proliferation, even in the presence of mitogens (Barres et al., 1994), suggesting that retinoids negatively regulate growth factor receptors such as PDGFRα, which has been shown in other tissues (Balmer and Blomhoff, 2002; Reiterer et al., 2008; Okada et al., 2012). In the *Raldh2* cKO mouse, therefore, it is possible that PDGF sensitivity persists in OPCs, thereby slowing differentiation.

In addition to a reduction in the number of CC1^+^ OLs, we observed decreased PLP signal in the CC of cKO mice between P14 and P120. This reduction is likely an effect of having too few OLs, but it may also reflect decreased *Plp* transcription. In C6 rat glioma cells, RA treatment upregulates *Plp* expression in a dose-dependent manner (Zhu et al., 1992). Moreover, in the *neckless* zebrafish mutant that lacks RALDH2, the expression of *Mbp*, another integral myelin protein, is reduced but can be rescued by exogenous RA (Kazakova et al., 2006). Despite the observed reduction in OLs and PLP, *Raldh2* cKO mice displayed no obvious behavioral abnormalities within the first month of postnatal life. It is possible that the number of OLs in cKO mice, albeit reduced, was sufficient to maintain neuronal function during this time.

### RALDH2 is necessary for neuron and white matter astrocyte development

We have shown that loss of RALDH2 decreases GFAP^+^ area in the postnatal CC, decreases the number of CTIP2^+^ and TBR1^+^ neurons in the layers 5 and 6, and increases the number of SATB2^+^ neurons in upper layers. In conjunction with the decrease in OLIG2^+^ cells in the CC, these results suggest that RALDH2 is involved in regulating the development of different cell types derived from NSCs in both embryonic and postnatal neurogenic and gliogenic structures (i.e., in the embryonic VZ and SVZ, the former of which is progressively lost during neurogenesis, and the postnatal SVZ) .

In *Raldh2* cKO mice, we observed a reduction in GFAP^+^ area between P2 and P21, a time during which local astrocyte proliferation increases astrocyte numbers as much as 8-fold, constituting >50% of cortical astrocytes, and astrocytes undergo morphologic maturation, in part due to rising GFAP expression (Bandeira et al., 2009; Ge et al., 2012). RA alone or in synergy with ciliary neurotrophic factor (CNTF) is able to increase the number of GFAP^+^ cells derived from late cortical progenitors *in vitro,* as well as increase *Gfap* expression (Faigle et al., 2008). RA synergizes with IL-6 family cytokines to relax chromatin around astrocyte genes and promote astrocytogenesis from NSCs (Asano et al., 2009; Herrera et al., 2010).

In addition to changes in OLC number and astrocyte development, we observed decreased numbers of CTIP2^+^ and TBR1^+^ deep layer neurons and increased numbers of SATB2^+^ neurons in the cortex of P2 *Raldh2* cKO mice. This is consistent with previous accounts of RA perturbations disrupting neuronal development (Siegenthaler et al., 2009; Choi et al., 2014; Haushalter et al., 2017). Siegenthaler et al., (2009) studied cortical neurogenesis in the hypomorph-null hybrid *Foxc1* mouse mutant, in which the dorsal forebrain meninges that strongly express RALDH2 fail to develop. *Foxc1* mutants displayed a defect in the balance between symmetric (self-renewing) and asymmetric (neurogenic) divisions, leading to a deficit in neurogenesis and decreased CTIP2^+^ and TBR1^+^ neurons. This could be partially rescued by exogenous RA and suggests that endogenous RALDH2 influences neurogenesis. In a different model of RALDH2 loss-of-function later in embryogenesis, early postmitotic neurons left the intermediate zone (IZ, the anlage of the CC) too rapidly and settled in the deep layers, leading to a surplus of deep layer neurons and a deficit in upper layer neurons [this study used a cytomegalovirus (*CMV*)*-β-actin -Cre^ERT2^:Raldh2^flox/flox^* mouse line and Tamoxifen was administered at E10; Haushalter et al., 2017], suggesting that RA signaling occurs in postmitotic migratory neurons to regulate migration and molecular identity. Furthermore, it has been shown that when a dominant negative RAR (dnRAR403) is introduced in the developing cortical plate during neurogenesis, neuroblasts only transiently express their appropriate cell markers before adopting neuronal identities from other layers and migrating to the layer corresponding to the new identity (Choi et al., 2014). A similar phenomenon may occur in the *Raldh2* cKO, such that deep layer neurons lose their layer 5 and 6 identities and take on those of the upper layers. Overall, our findings suggest that RA may play multiple roles in neuronal development, as well as in glial development and myelination.

## Synthesis

Reviewing Editor: Orly Reiner, Weizmann Institute of Science Decisions are customarily a result of the Reviewing Editor and the peer reviewers coming together and discussing their recommendations until a consensus is reached. When revisions are invited, a fact-based synthesis statement explaining their decision and outlining what is needed to prepare a revision will be listed below. The following reviewer(s) agreed to reveal their identity: Julie Siegenthaler.

Retinoic acid (RA) is well-studied developmental signal for the CNS. However its role in OPC development and maintenance has not been rigorous explored nor do we know all the important sources of RA in the developing brain. This work advances the field as it identifies a previously unknown role for RA production by Ng2-lineage cells in regulation of OPC production and differentiation into OL during development and identifies that RA is potentially regulating Shh signaling in OPCs.

Specific comments:

The authors should integrate discussion on previous work (see below) and remove strong comments e.g. “Our work suggests for the first time a role for RALDH2-dependent RA synthesis by NG2+ cells in OPC production”.

Previous studies include: Mey et al.,2005; and the follow on papers Goncalves et al., 2018, 2019. In the 2019 paper (Goncalves et al) knock out of raldh2 was shown in NG2+ cells and OPC differentiation prevented. Thus some of the data presented is confirmatory and this needs to be acknowledged.

1. Ln 76-78 In the intro, the authors should specify that the meninges and perivascular Collagen1+ (Col1+) cells appear to be the main cells that express Raldh2 in the postnatal and adult forebrain, based on the literature. There are other Raldh2-expressing neuronal populations in the CNS so should not refer to CNS as a whole in this sentence. With regard to Ng2, OPCs of course are well described to express Ng2 however the Kelly et al., 2016 paper did not look at Ng2 in the meninges or the Col1+ perivascular cells in the brain parenchyma. It's important to show that Ng2 is expressed by these cells or that they are recombined by the Ng2-Cre (could be they express Ng2 at some point in their lineage, may no longer have Ng2 protein expression).

2. In general, the use of the term 'vascular/leptomeningeal cells' is confusing and likely inaccurate. Is this referring to all perivascular cells (pericytes, vSMCs and Col1+ perivascular cells)? This needs to be much better explained, also citing the literature showing which perivascular cells do or do not express Ng2 as well as what has been described previously for the Ng2-Cre in terms of recombination.

2. Pdgfrb and Ng2 cells described in Fig. 2 are pericytes, these are well known to express both of these proteins and associate with the brain microvasculature. These are a different population than the Col1-expressing perivascular cells previously shown to express Raldh2 (Kelly et al., 2018) and described in recent scRNA-seq data sets on the cerebral vasculature (Vanlandewijck et al., 2018 Nature). Further, the Raldh2/Pdgfrb immunostaining is also consistent with Raldh2 in pericytes. This hasn't been described in other studies of Raldh2 protein and gene expression in the postnatal brain (Wagner et al., 2002 Cerebral Cortex and Smith et al., 2001 Cerebral Cortex)) and it is a concern that the author's do not see Raldh2 mRNA signal in a pattern that would indicate expression in pericytes. This raises the possibility that the antibody detection of Raldh2 is not completely reliable. Is there evidence from recent single cell data sets (Vanlandewijck et al., 2018) that brain pericytes actually express Raldh2?

2. Raldh3 is expressed in the ventral forebrain developmentally and into postnatal timepoints and Raldh1 is expressed in the striatum postnatally as well as the postnatal meninges (please see Smith et al and Wagner et al papers). It's surprising that given proximity of these areas of Raldh1 and Raldh3 expression to the SVZ, it is not sufficient to compensate for deletion of Raldh2 in a limited population of cells. It would be important for the authors to comment on potential reasons for this in their discussion.

3. The author's show there is a reduced number of deep layer neurons (Ctip2, Tbr1) in the Raldh2 cKO mice however they state there is no reduction in cortical thickness. Quantification needs to be performed, low mag DAPI images are not sufficient to make this claim. Further, if the cortical thickness is not sig. different then what is compensating for the loss of lower layer neurons? Are other layers expanded?

4. For all of their images where the individual signal is depicted, they need to provide the overlay so the reader can evaluate the cell co-localization.

5. Need a citation for these statements in Ln 276-279, or provide data to support this.
